# Surgically Treated Non-Unions and Fractures of the Femur and Tibia in Adults: A Nationwide Analysis of 888,442 Cases from the German InEK Database

**DOI:** 10.1007/s44197-026-00545-8

**Published:** 2026-03-29

**Authors:** Niklas Graefe, Ludwig Matrisch, Sebastian Findeisen, Tobias Grossner, Jan Streblow, Thomas Ferbert, Gerhard Schmidmaier, Tim Niklas Bewersdorf

**Affiliations:** 1 Heidelberg Trauma Research Group, Clinic for Trauma and Reconstructive Surgery, Centre for Orthopaedics, Trauma Surgery and Spinal Cord Injury, Heidelberg University Hospital, Heidelberg, 69120 Germany; 2 Faculty of Medicine, Heidelberg University, Heidelberg, 69120 Germany; 3 Medical Clinic I, University Hospital Schleswig-Holstein, Lübeck, Germany

**Keywords:** Non-union, Fracture, Nationwide database study, Non-union/fracture ratio, Femur, Tibia

## Abstract

**Background:**

Non-unions (NU) after fractures, especially in cases with the need of surgical intervention, represent one of the most severe complications in traumatology. As previous studies estimate post-fracture NU risk between 2% and 10% data inconsistencies persist. In particular, the data on the influence of different anatomical regions, age and sex on fracture associated NU is scarce. Therefore, this study aims to provide a comprehensive descriptively assessment of the ratio of surgically treated tibia and femur NU to tibia and femur fractures based on a nationwide dataset, with a specific focus on differences across anatomical regions.

**Methods:**

This retrospective, population-based, cross-sectional analysis of the German InEK database (2019–2023) was conducted, including 888,442 fractures and 3,017 NU cases. The study focused on ICD- (International Statistical Classification of Diseases and Related Health Problems-) coded diagnoses of femur and tibia NU and their associated fractures. To enhance precision, fracture and NU sites were grouped according to different anatomical regions assessed by documented OPS- (Operation and Procedure Classification System) codes. Subgroup analyses were performed for age and sex.

**Results:**

Surgically treated femoral shaft NU had the highest incidence (0.18 cases per 100,000 residents and year), followed closely by tibial shaft NU (0.16). The highest NU/100-fractures ratio was observed in tibial shaft (1.277), followed by distal tibia (1.095) and distal femur (0.996). Across all localisations, men had clearly higher NU/100-fractures ratios than women. NU/100-fractures ratios also varied with age, with highest ratio in the economically active population, followed by a generally decline in older patients.

**Conclusion:**

This nationwide database study indicates that surgically treated NU/fracture ratios of femur and tibia are generally low. The proportion of NU to fracture differ between different anatomical regions. In general, proximal localisations showed lower NU/100-fractures ratios than distal localisations within the femur and in comparison of femur and tibia. Independently of the anatomical localisation men had a higher NU/100-fractures ratio than women. As highest NU/100-fractures ratios were detected in the economically active population, this highlights the substantial socioeconomic burden associated with this condition.

## Background

Non-unions (NU) present a huge challenge in modern traumatology, as they pose a great healthcare and patient burden. The European Society of Tissue Regeneration in Orthopaedics and Traumatology (ESTROT) defines a NU as a condition in which the treating physician observes no further signs of healing, indicating that the fracture will not heal without additional intervention [1, 2]. According to AO Foundation a NU is a condition, in which bone healing has stopped and consolidation needs a surgical intervention [1]. This necessitates targeted and precise therapeutic measures, such as re-osteosynthesis, debridement of sclerotic or necrotic bone adjacent to the NU site, treatment of a potential infection and bone grafting [1]. Furthermore, in clinical practice, NU is often diagnosed when bone healing fails to occur within six to nine months [3, 4]. Due to differences in NU definitions, periods between fracture and NU diagnosis varies from three up to twelve months in literature [3–5].

NU therapy is complex and in most cases several surgeries are necessary resulting in healing rates ranging from 77% in large size bone defects up to > 90% in smaller defects [2, 6, 7]. Particularly, NU of the lower extremity represents a severe disruption to daily life. The limited mobility typically affects not only the patient but also their relatives and due to prolonged time to consolidation of up to 16 months return to work is commonly delayed [8]. Therefore, NU of the lower extremity possesses a huge socio-economic burden [9, 10]. The risk of post-fracture NU has already been determined by numerous studies, but the results vary considerably, ranging from 2% to 10%, depending on the anatomical localisation and the study [11–15].

As population-based estimates are strongly influenced by study design, case definitions, and the level of anatomical resolution, reported rates of fracture-related NU vary widely across the literature. Many registry-based analyses rely exclusively on ICD (International Statistical Classification of Diseases and Related Health Problems) codes. These codes provide only limited information about anatomical localisation and, in some instances, group multiple bones within the same category. This restricts the ability to assess heterogeneity within individual long bones.

Against this background, the present retrospective, population-based, cross-sectional, register-based study was conducted to assess the population-level burden of surgically treated NU following femur and tibia fractures. In addition, NU/fracture ratios were analysed with stratification by anatomical region, age, and sex. To improve anatomical resolution beyond ICD-based classifications, we combined ICD-diagnosis codes with OPS- (Operation and Procedure Classification System) codes, enabling a more detailed approximation of specific regions within the femur and tibia than previously reported [11, 14, 15]. While the design does not allow individual-level risk estimation or causal inference, it provides detailed insights into anatomical patterns of surgically managed NU in a high-income country.

## Methods

### Data Acquisition

The aim of this study was to descriptively assess the incidence of surgically treated NU and ratio of surgically treated NU to fractures of a specific anatomical region of the femur and the tibia at an entire population-level with a further stratification according to age and sex. We combined ICD- and OPS-codes to achieve a more detailed insight into differences of specific anatomical regions. The InEK database (InEK Daten Browser; Institut für das Entgeltsystem im Krankenhaus GmbH (InEK), Siegburg, Germany: https://datenbrowser.inek.org/) is intended to create a meaningful and reliable information basis ‘for a well-founded and objective review of the effects of the measures adopted with the COVID-19 Hospital Relief Act’ [16]. The InEK database was initiated by statutory and private health insurance providers and the German Hospital Federation. Since it is based on the Diagnosis-Related Groups (DRG) system, which serves as the national framework for case-based hospital reimbursement, the register provides anonymized data for analysis of all medical aspects beyond COVID-19 [16]. The DRG statistics of the InEK data browser are characterized by an annual survey of inpatient cases from all German hospitals [17]. In addition to patients’ age and sex, the data includes all ICD-coded diagnoses of all inpatients based on the ICD-10 as well as all surgical procedures and interventions, coded by OPS-codes, which were performed during inpatient treatment.

The evaluation period of five years began on January 1st, 2019, which marks the start of InEK data availability and ended on December 31st, 2023. Initially, a query of coded diagnoses for surgically treated NU (3,017 cases) and fractures (888,442 cases) was performed, with a detailed analysis based on patient sex and age, categorized into distinct age groups. For this analysis, two anatomical localisations of surgically treated NU were selected, identified by the ICD-codes M84.15 (fracture-related NU of pelvic region and thigh) and M84.16 (fracture-related NU of lower leg). These two anatomical regions were further subdivided into seven additional subregions using OPS-codes. To calculate incidences of fractures and NU per 100,000 residents, we used official data reported by the German Federal Statistical Office on the adult population in Germany during the corresponding time period [18].

The ICD-coded diagnoses of surgically treated NU from the various anatomical localisations (Table 1) were then combined in a subsequent query with an OPS-coded surgical procedure (OPS-code 5-782 including its subcodes, as listed in Table 2). As NU definitions vary in time periods between fracture and NU diagnosis from three up to twelve months [3–5], it is not possible to determine a specific time interval between analyses of documented fractures and NU. Therefore, we assessed fractures (denominator) and NU occurrences (numerator) within the same period, although NU and fracture cohorts were not identical. Due to the database characteristics, an individual tracking of fracture and NU patients was not possible. Consequently, this study was designed retrospectively, population-based and cross-sectional. Therefore, it reports NU/100-fractures ratios instead of exact risks and the ratio should be interpreted as a system-level indicator rather than as the individual risk of NU after fracture. All patients aged < 18 years were excluded from the study.

**Table 1:** Included ICD-codes and referring fracture types

ICD-Code	Description
S72.0	Fracture of head and neck of femur
S72.1	Pertrochanteric fracture
S72.2	Subtrochanteric fracture of femur
S72.3	Fracture of shaft of femur
S72.4	Fracture of the distal femur
S82.1	Fracture of the proximal tibia
S82.2	Fracture of shaft of tibia
S82.3	Fracture of the distal tibia

**Table 2:** Included OPS-codes and referring non-union types

OPS-Code	Non-union type
5-782.*e	Non-union of the head and neck of femur
5-782.*f	Non-union of the proximal femur
5-782.*g	Non-union of the shaft of femur
5-782.*h	Non-union of the distal femur
5-782.*k	Non-union of the proximal tibia
5-782.*m	Non-union of the tibia shaft
5-782.*n	Non-union of the distal tibia

**Fig. 1:**
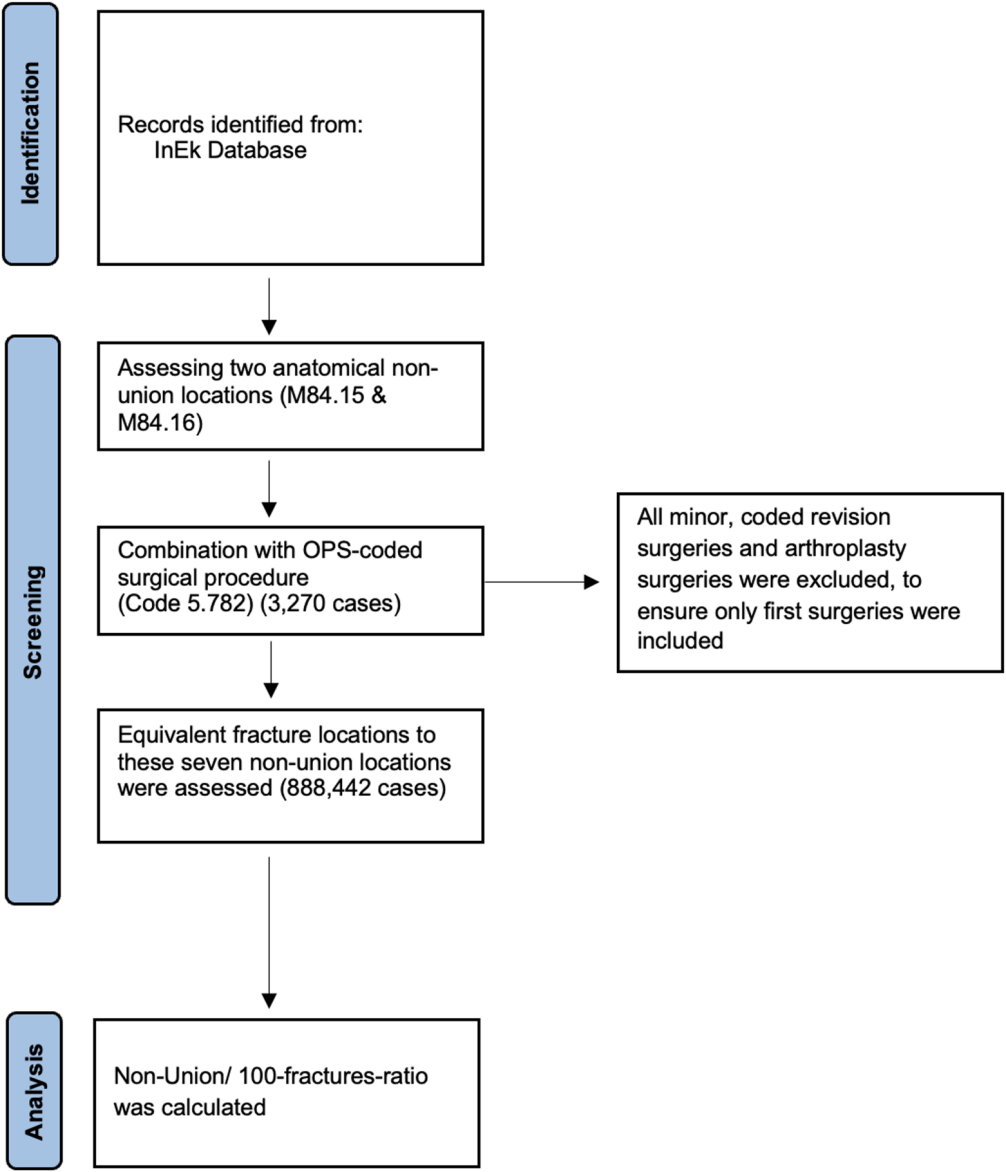
Study design and data acquisition. The flowchart outlines the process for identifying and analysing fracture-related non-union cases from the InEK database. It consists of three main stages: identification, screening, and analysis

Since the case-based system could result in duplication of cases, all coded revision surgeries were excluded by filtering for the OPS-code 5-782 to ensure only initial surgeries were included. This approach aimed to capture only newly diagnosed surgically treated NU and allowed further subdivision of the regions into proximal, shaft, and distal sections of femur and tibia, as well as femoral head and neck. As femoral head and neck fractures are frequently treated with total hip arthroplasties and NU formation after arthroplasty is not possible, all fractures treated with arthroplasty surgeries were excluded from the study. Equivalent to these seven anatomical localisations of NU, fractures in the corresponding anatomical regions were also queried to calculate the NU per fracture ratio (Table 1). Detailed information about the study design is displayed in Fig. 1.

The InEK database provides data divided into 10-year age intervals up to the age of fifty, followed by 5-year age intervals for patients aged between 50 and 79 years old. Elderly patients aged 80 years or older are consolidated by the InEK database into one group. Besides age-dependent differentiation, patients were differentiated by sex for further analysis. Due to the data protection policy of the InEK database a combined differentiation of sex and age-groups was not possible.

### Statistics

Since the InEk database provide information about all cases recorded in the national DRG database of Germany of the years 2019 to 2023, we included the entire adult population, and no sampling was performed [19]. NU/fracture ratios were calculated as the number of documented NU divided by the number of documented fractures per stratum (sex, age group, anatomical localisation), expressed as NU/100-fractures ratio. Sex- and age-specific differences were assessed using Poisson regression models with the number of NU cases as the dependent variable, log(fracture number) as offset, and sex or age group as categorical predictors. Exponentiated regression coefficients represent rate ratios (RR) with 95%-confidence intervals (95%-CI). The youngest age group (18–29 years) served as reference category. All analyses were performed using R version 4.5.2 (The R Project for Statistical Computing, Vienna, Austria). Statistical significance was defined as *p* < 0.05. Further analyses were performed using the Python-based tool Matplotlib 3.8.0 (https://matplotlib.org/3.8.0/index.html) and Microsoft Office Version 2506 (Redmont, WA, USA) [19]. Figures were also created by Matplotlib 3.8.0.

## Results

**Fig. 2:**
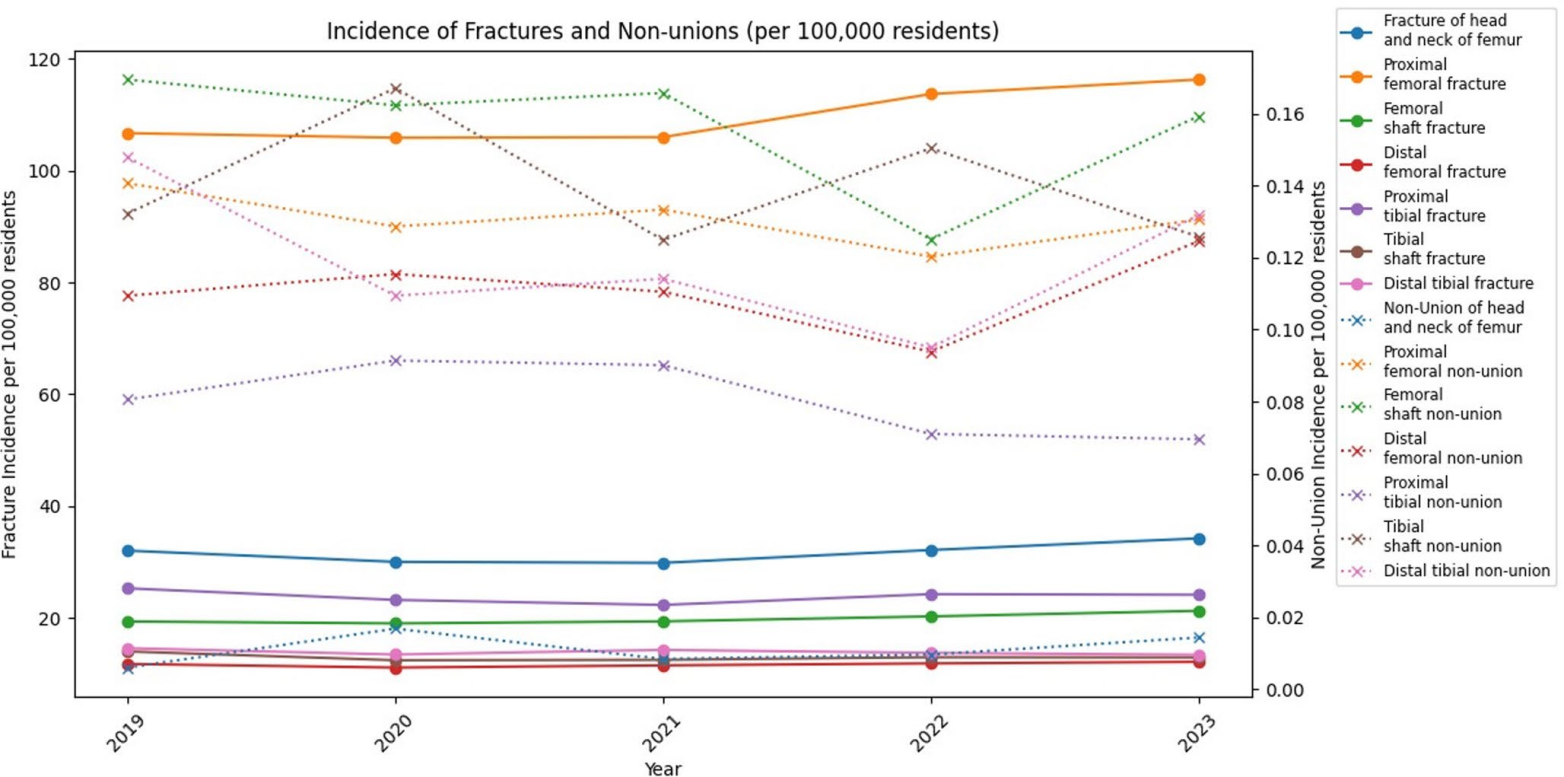
Incidence per 100,000 residents of non-unions and fractures of femur and tibia divided by year of diagnosis

In total, 888,442 femur and tibia fractures were documented in the InEK database during the study period. Proximal femur fractures were the most frequent, with an average incidence of 109.10 cases per 100,000 residents annually. The second most common group consisted of non-prosthetically treated femoral head and neck fractures, with an annual incidence of 31.27 cases per 100,000 residents. The remaining five fracture categories ranged between 10 and 23 cases per 100,000 residents annually. A slight decline in the incidence of almost all fracture types was observed during COVID-19 restrictions in 2020 and 2021. In total, the data indicates an average of 212.27 fractures of the femur and tibia per 100,000 residents annually. Absolute numbers are displayed in Table 3, while incidences are visualized in Fig. 2.

**Table 3:** Absolute case numbers of femur and tibia fractures divided by anatomical region and year of diagnosis

Fracture Type	2019	2020	2021	2022	2023	Average per year
Fracture of head and neck of femur	26,477	24,814	24,672	26,565	28,387	26,183
Proximal femur fracture	88,518	87,853	89,025	94,309	96,850	91,311
Femur shaft fracture	14,245	14,008	14,347	15,154	15,964	14,744
Distal femur fracture	9,211	8,706	9,022	9,337	9,643	9,184
Proximal tibia fracture	19,696	18,224	17,527	18,979	18,958	18,677
Tibia shaft fracture	9,400	8,337	8,280	8,757	8,752	8,705
Distal tibia fracture	9,266	8,628	9,124	8,881	8,526	8,885
Total	185,313	177,670	181,047	188,982	185,180	185,638

While 888,442 femur and tibia fractures were documented during the study period, only 3,017 surgically treated NU were reported. The annual analysis of absolute numbers of newly diagnosed surgically treated NU between 2019 and 2023 revealed only minor differences. However, notable differences were observed depending on the specific anatomical localisation. The highest incidence was recorded for surgically addressed femoral shaft NU, with an average of 0.18 cases per 100,000 residents annually, followed by tibial shaft NU with an incidence of 0.16 cases (Table 4; Fig. 2). Proximal femur NU requiring surgical intervention accounted for an average of 0.15 cases per 100,000 residents annually. Distal tibial NU accounted for 0.14 cases and distal femur NU for 0.13 cases per 100,000 residents annually. The lowest rates were observed for surgically treated proximal tibial NU, averaging 0.09 cases per 100,000 residents annually. Surgically treated femoral head and neck NU were even less frequent, with only 0.01 cases per 100,000 residents annually. Notably, except for surgically addressed tibial shaft NU, a decline in case numbers was observed in 2022, followed by a return to baseline levels in 2023. Overall, this corresponds to an average of 0.86 newly diagnosed NU cases, which underwent surgical interventions, per 100,000 residents annually affecting the femur and tibia.

**Table 4:** Absolute numbers of surgically treated non-unions after femur or tibia fracture divided by year of diagnosis

Non-union type	2019	2020	2021	2022	2023	Average per year
Head and neck of femur	5	12	7	8	11	8,6
Proximal femur	117	105	110	93	105	106
Shaft of femur	139	131	138	102	132	128,4
Distal femur	89	95	92	78	103	91,4
Proximal tibia	65	73	73	54	55	64
Shaft of tibia	104	132	99	119	100	110,8
Distal tibia	120	79	92	74	106	94,2
Total	639	627	611	528	612	603,4

The proportion of revision surgeries, which were coded by the OPS-code 5-782, varied across anatomical localisations. NU of femoral head and neck fractures had the lowest revision rates of < 0.1%, followed by surgically treated proximal femoral NU with revision rates of 7.5%. Surgery of distal femoral (10.6%), distal tibial (11.0%), and femoral shaft NU (11.8%) resulted in similar revision frequencies. The highest average revision rates were observed in tibial shaft NU at 14.2% and proximal tibia NU at 15.8%. As mentioned, all coded revision surgeries were excluded from the analysis to determine the exact occurrence of surgically treated NU and calculating the NU/100-fractures ratios.

**Fig. 3:**
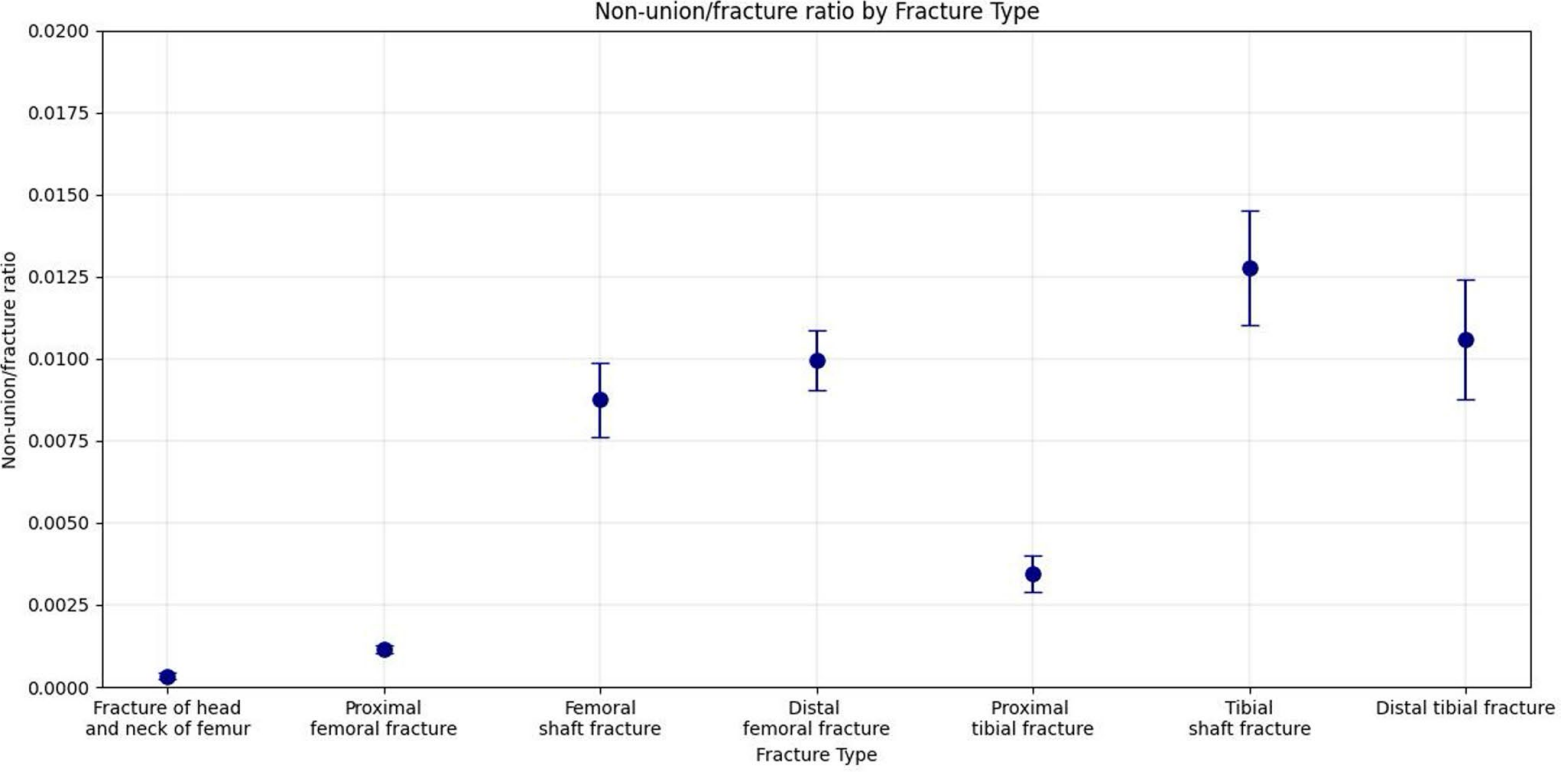
Ratio of non-unions compared to fractures of the same anatomical localisation. The x-axis represents the specific fracture sites. Dots show the mean; error bars show the error of the means

When comparing the numbers of NU with fractures of the same anatomical localisation, clear differences become apparent, reflecting the ratio of NU compared to fractures (Table 5). Surgically treated femur shaft NU, despite being the most frequent in absolute numbers, ranks only fourth in terms of NU/100-fractures ratio (0.875). The highest NU/100-fractures ratio was observed for the tibial shaft, with a ratio of 1.277, followed by the distal tibia (1.095) and the distal femur (0.996) (Table 5 and Fig. 3). Therefore, NU/100-fractures ratio of tibia shaft was 38.7-times higher than the observed ratio of femoral head and neck and 11.0-times higher than the observed ratio of the proximal femur.

**Table 5:** Non-union/100-fractures ratio of the femur and tibia divided by anatomical localisations. NU: non-union, 95%-CI: 95%-confidence interval. Standard deviation between the years 2019 to 2023

Anatomical localisation	NU/100-fractures ratio	Standard deviation	95%-CI
Femoral head and neck	0.034	0.009	0.011–0.055
Proximal femur	0.116	0.012	0.094–0.138
Shaft of femur	0.875	0.113	0.720–1.022
Distal femur	0.996	0.091	0.791–1.199
Proximal tibia	0.344	0.055	0.259–0.427
Shaft of tibia	1.277	0.176	1.036–1.510
Distal tibia	1.095	0.181	0.846–1.274

**Fig. 4:**
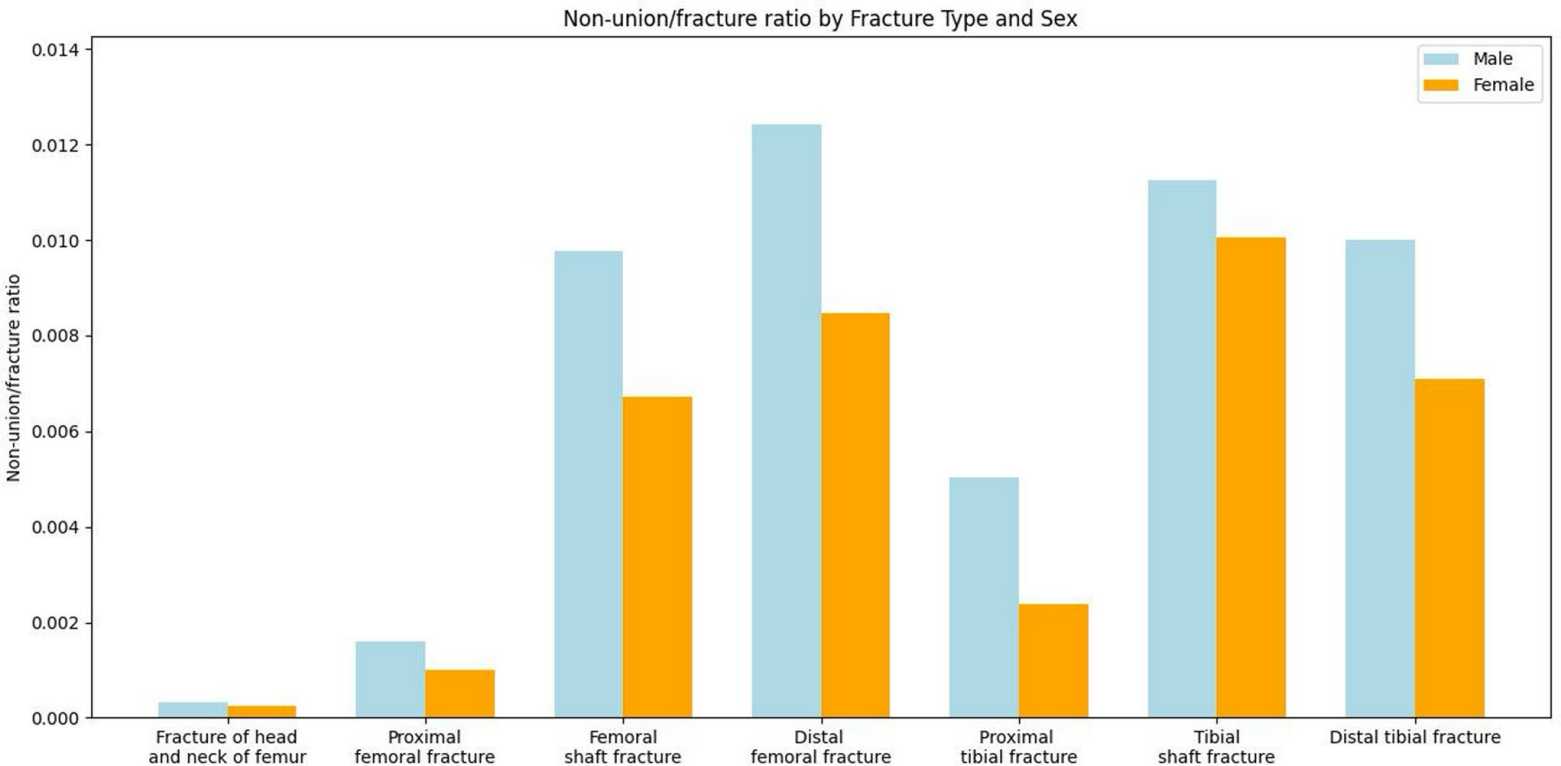
Ratio of non-unions compared to fractures of the same anatomical localisation divided by sex. The x-axis represents specific fracture sites

Analyses of sex-specific NU/100-fractures ratios reveal that men had a higher NU/fracture ratio across all localisations (men versus (vs.) women: 0.431 vs. 0.256 per 100 fractures; RR: 1.66, 95%-CI: 1.55–1.78, *p* < 0.001), as displayed in Fig. 4; Table 6. Calculated RR showed the greatest disparities between males and females in the proximal tibia (RR (men vs. women): 2.15; 95%-CI: 1.73–2.67, *p* < 0.001), followed by the proximal femur (RR (men vs. women): 1.58; 95%-CI: 1.33–1.88, *p* < 0.001) and the distal femur (RR (men vs. women): 1.46; 95%-CI: 1.21–1.78, *p* < 0.001). While highest NU/100-fractures ratio in men was detected for the distal femur (1.241 NU/100-fractures), highest NU/100-fractures ratio in women was calculated for tibia shaft (1.006 NU/100-fractures).

**Table 6:** Sex-specific NU/100-fractures ratios of different anatomical localisations. NU: non-union, 95%-CI: 95%-confidence interval

Anatomical localisation	NU/100-fractures ratio in males	NU/100-fractures ratio in females	Rate ratio	95%-CI	*p*-value
Femoral head and neck	0.034	0.024	1.39	0.78–2.49	0.265
Proximal femur	0.159	0.101	1.58	1.33–1.88	**< 0.001**
Shaft of femur	0.977	0.672	1.45	1.25–1.70	**< 0.001**
Distal femur	1.241	0.848	1.46	1.21–1.78	**< 0.001**
Proximal tibia	0.503	0.234	2.15	1.73–2.67	**< 0.001**
Shaft of tibia	1.126	1.006	1.12	0.95–1.32	0.190
Distal tibia	1.000	0.708	1.41	1.18–1.69	**< 0.001**
Overall	0.431	0.256	1.66	1.55–1.78	**< 0.001**

**Fig. 5:**
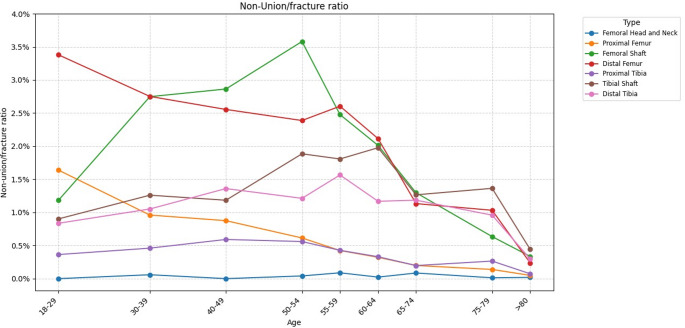
Ratio of non-unions compared to fractures divided by fracture type and age. The y-axis represents the non-union/fracture ratio, while the x-axis displays the age groups

The NU/100-fractures ratios varied depending on age, as shown in Fig. 5 and Table 7. The highest NU/100-fractures ratio was found for femoral shaft localisations of patients aged 50–54 years with a ratio of 3.58 NU per 100 fractures, followed by the distal femur in patients aged 18–29 with a ratio of 3.38 NU per 100 fractures. The overall RR for NU/100-fractures ratio was highest in the 40–49 years old age group with RR of 1.18 (95%-CI: 1.02–1.38; p = 0.030). With further increasing age the overall RR decreased continuously with lowest RR in patients aged ≥ 80 years (RR: 0.09; 95%-CI: 0.08–0.10; p < 0.001). In detail, we detected for every anatomical localisation that in the oldest age group the RR was the lowest, reflecting that the NU/100-fractures ratio in this cohort was also the lowest. Femur and tibia shaft, as well as proximal and distal tibia NU/100-fractures ratios showed bell-shaped configurations with first increasing NU/100-fractures ratios with increasing age in younger age groups, followed by decreasing NU/100-fractures ratios with increasing age in older age groups. In contrast to this, we detected for the proximal and distal femur continuously deceasing NU/100-fractures ratios with increasing age. Therefore, RR of all age groups in comparison to the age group “18–29” was < 1 with a decreasing RR with increasing age. As NU/100-fractures ratio of femoral head and neck fractures in the reference age group “age 18–29” was 0, no inferential statistics for this anatomical localisation were performed.

**Table 7:** Age-specific rate ratio of NU/100-fractures of different anatomical localisations. 95%-CI: 95%-confidence interval

Anatomical localisation	Rate ratio, [95%-CI] and *p*-value in comparison to reference age group (18–29 years)							
	30–39	40–49	50–54	55–59	60–64	65–74	75–79	≥ 80
Proximal femur	0.56 [0.30–1.03] *p* = 0.063	0.56 [0.32–0.96] *p* = 0.034	0.38 [0.22–0.66] *p* < 0.001	0.27 [0.16–0.46] *p* < 0.001	0.20 [0.12–0.33] *p* < 0.001	0.12 [0.08–0.20] *p* < 0.001	0.08 [0.05–0.13] *p* < 0.001	0.03 [0.02–0.05] *p* < 0.001
Shaft of femur	2.30 [1.61–3.27] *p* < 0.001	2.50 [1.75–3.58] *p* < 0.001	2.69 [1.89–3.84] *p* < 0.001	2.06 [1.45–2.92] *p* < 0.001	1.69 [1.20–2.38] *p* = 0.003	1.04 [0.77–1.42] *p* = 0.784	0.53 [0.37–0.76] *p* < 0.001	0.28 [0.20–0.37] *p* < 0.001
Distal femur	0.98 [0.65–1.50] *p* = 0.937	0.76 [0.50–1.15] *p* = 0.193	0.71 [0.46–1.08] *p* = 0.111	0.81 [0.57–1.17] *p* = 0.260	0.65 [0.45–0.93] *p* = 0.020	0.34 [0.24–0.48] *p* < 0.001	0.29 [0.20–0.43] *p* < 0.001	0.08 [0.05–0.11] *p* < 0.001
Proximal tibia	1.34 [0.80–2.26] *p* = 0.267	1.97 [1.22–3.16] *p* = 0.005	1.95 [1.21–3.15] *p* = 0.006	1.33 [0.82–2.16] *p* = 0.248	1.10 [0.66–1.82] *p* = 0.719	0.53 [0.31–0.92] *p* = 0.023	0.74 [0.40–1.37] *p* = 0.336	0.19 [0.09–0.41] *p* < 0.001
Shaft of tibia	1.44 [1.07–1.95] *p* = 0.016	1.52 [1.13–2.05] *p* = 0.005	2.21 [1.63-3.00] *p* < 0.001	2.14 [1.58–2.89] *p* < 0.001	2.15 [1.56–2.95] *p* < 0.001	1.47 [1.07–2.03] *p* = 0.019	1.72 [1.13–2.62] *p* = 0.011	0.54 [0.34–0.86] *p* = 0.009
Distal tibia	1.26 [0.87–1.84] *p* = 0.221	1.62 [1.14–2.30] *p* = 0.007	1.48 [1.01–2.18] *p* = 0.045	1.94 [1.36–2.78] *p* < 0.001	1.44 [0.98–2.13] *p* = 0.066	1.35 [0.93–1.94] *p* = 0.113	1.21 [0.74–1.98] *p* = 0.439	0.32 [0.18–0.56] *p* < 0.001
Overall	1.15 [0.98–1.35] *p* = 0.079	1.18 [1.02–1.38] *p* = 0.030	1.09 [0.94–1.28] *p* = 0.259	0.97 [0.83–1.13] *p* = 0.681	0.72 [0.61–0.84] *p* < 0.001	0.43 [0.37–0.49] *p* < 0.001	0.27 [0.23–0.32] *p* < 0.001	0.09 [0.08–0.10] *p* < 0.001

## Discussion

As risk rates of NU formation after fracture vary in literature between 2 and 10%, there is a distinct need for further research about determination of NU occurrence, especially in comparison to fracture occurrences [11–15]. Studies examining fracture-related NU with detailed differentiation of exact anatomical regions remain scarce and are often limited to smaller cohorts or selected clinical populations. Using nationwide administrative data covering the entire adult population of Germany, we analysed more than 800,000 femur and tibia fractures and over 3,000 cases of surgically treated NU over a five-year period. By combining ICD- and OPS-codes, this study offers a more detailed anatomical perspective than ICD-based analyses alone. Consequently, this study reveals a substantial heterogeneity between different anatomical localisations within the same long bone in a high-income country.

When comparing our findings with the existing literature, the calculated NU/100-fractures ratios for the femur and tibia in our study appears to be at the lower end of previously reported values. This is likely because the present analysis included only surgically treated NU, whereas other studies also considered conservatively managed cases. Other register-based studies reported that NU risk after fracture varies between 2 and 5% [11, 14]. Most register-based studies share the same limitation of imprecisely determination of the anatomical localisation of the NU. While ICD-codes for fractures clearly describe the anatomical localisation, ICD-codes of NU provide solely unspecific anatomical information, like “pelvis and femur” and “lower leg”. Therefore, the approach of calculation NU risk based on ICD-codes only result in imprecisely anatomical resolution. On the other side, OPS-codes used for NU surgery clearly identify the anatomical region but are limited to surgically treated cases only. Consequently, the combination of ICD-codes and OPS-codes not only provides information about which bone is affected (pelvis or femur; tibia or fibula), but also specifies the exact anatomical region, like proximal, shaft or distal [11, 14]. Although using OPS-codes excluded all conservatively treated NU, this approach provides an exceptional detailed insight into differences between different anatomical localisations of the same bone. As the strength of the present study is the high anatomical resolution, which was achieved by focusing exclusively on surgically treated NU, this might result in a potential slight underestimation of overall NU occurrence.

A study by Reeh et al. offers the greatest comparability, as it is also a register-based study based on ICD-codes. It focused on the same NU localisations and also included the total population of Germany. Because Reeh et al. localised NU based solely on ICD codes, they reported NU/fracture ratios for the upper leg (ICD: M84.15) and the lower leg (ICD: M84.16). In their analysis, these ratios were slightly higher than the corresponding NU/fracture ratios observed in our study. These marginally higher ratios can be attributed to the key difference that the study of Reeh et al. did not exclude revision surgeries but included conservatively treated or asymptomatic patients [11]. According to our results surgical revisions of femur and tibia NU account for 7.5% to 15.8%, which were subtracted in our study. This is consistent with the literature, which reports a consolidation rate of approximately 90% in NU cases of the lower extremity [20, 21]. Additionally, the case-based system in Germany may generate false high numbers of documented NU, because the DRG-system allows up to four cases per year to be attributed to a single patient. Many NU are treated in multiple stages in different quarters of the year (e.g., using the induced membrane technique [22]), which can distort the data. In our study such a potential duplication bias was eradicated by counting only the combination of NU and initial surgery as a single case. Not excluding these revisions would lead to an overestimation of NU diagnoses. Another important difference is that Reeh et al. divided the lower extremity into three sections based on the ICD-system (hip and thigh; lower leg; ankle and foot), whereas we analysed NU formation for specific anatomical regions (proximal, shaft, and distal) of femur and tibia. Consequently, Reeh et al. also included NU of the pelvis and the fibula, which can also modify NU occurrences after fracture [22].

A study published by Mills et al. examined the risk and incidence of NU after fracture in the Scottish population and was also based on ICD-coded diagnoses [14]. As this study also used ICD-codes for NU diagnoses, the anatomical localisation of the NU was imprecisely, too. While we detected NU/100-fractures ratios up to 1.277, Mills et al. reported a risk of 5.4% for the lower leg [14]. Even the subgroup analysis of the age group with the highest NU/100-fractures ratio of the tibia shaft showed a ratio of 1.976, which was also lower than reported by Mills [14]. A plausible reason for the discrepancy could be that fibula NU were not reported in our study, as proximal and shaft NU of the fibula are often treated conservatively. Consequently, an OPS-guided approach, as we used, is not suitable to detect fibula NU properly. Furthermore, Mills et al. mentioned that in some cases multiple surgical procedures could be counted as multiple NU resulting in higher incidences. After elimination of this error, they reported an overall NU risk after fracture of approximately 1.7%, which is only slightly higher than the cumulative NU/100-fractures ratio in our study [14]. Both the study by Reeh et al. and Mills et al. reported all diagnosed NU including both surgically and conservatively treated NU, while this study focused on surgically treated NU only [11, 14, 23, 24]. Ultimately, this resulted in a lower NU/100-fractures ratio in our study.

The considerable differences in NU/fracture ratios of different anatomical localisations can be explained by local physiologic factors. It is well-known that regions near the joints of long bones have a better arterial blood supply compared to shaft regions [25–27]. This results in lower NU/fracture ratios of the proximal and distal tibia compared to tibia shaft and lower NU/fracture ratios of the proximal femur compared to femoral shaft. As distal femur fractures are often a consequence of high-energy trauma with relevant dislocation this results frequently in open fractures and impaired blood supply [28–30]. Consecutively, NU/100-fractures ratio in the distal femur is high and comparable to NU/100-fractures ratio of the femur shaft. Furthermore, soft tissue coverage plays a crucial role in successful bone healing [4, 31, 32]. The tibial shaft has less soft tissue coverage, resulting in reduced blood supply and making it more susceptible to open fractures and consecutive bone infection [4, 32]. On the other side, due to low-energy trauma, good soft tissue coverage, and almost no open fractures, the detected NU/100-fractures ratio of the femoral head and neck as well as the proximal femur was exceptionally low. Explaining the findings of this study by pathophysiological findings within the literature, the higher NU/100-fractures ratio of tibial localisations could be in general a consequence of higher rates of open fractures, worse blood supply and less tissue coverage of the lower leg compared to the upper leg [2, 15, 33]. Nevertheless, as an individual case-tracking within the InEK database is not possible, these potential explanations should be confirmed in future studies.

Analysis of documented fractures per year shows lower fracture incidences in 2020 and 2021. This reduction was most likely related to the COVID-19 pandemic and associated governmental lockdowns and restrictions and is consistent with findings reported by Heinz et al. [34]. In March 2022 most of the governmental restrictions were withdrawn and therefore fracture incidences rose again in 2022. Assuming surgically treated NU/fracture ratios remained constant during COVID-19 lower fracture incidence led to lower NU incidence, as it was reported in 2021 and 2022 (except for tibial shaft NU). Due to time after trauma as a diagnostic criterion for NU, decreased NU numbers were documented several months after lower fracture numbers occurred. Therefore, calculated NU/100-fractures ratios in 2022 pretend to be lower but are in fact a consequence of lower fracture incidence in 2021.

When considering the overall NU after fracture ratio in the male vs. female population, our data shows that males have a significantly higher overall NU/100-fractures ratio. In detail, except in femoral head and neck as well as tibia shaft localisations, the male sex was associated with a significantly higher NU/100-fractures ratio than the female sex. According to other studies male patients sustain more often high-energy trauma like sports or vehicle accidents than women resulting in higher percentage of severe fracture dislocation or open fractures and therefore higher NU/100-fractures ratios. Besides trauma associated risk factors, there exist several patient associated risk factors, like smoking or diabetes, which compromise bone healing and therefore promote NU formation. Both smoking and diabetes are more common in males and could also increase NU/100-fractures ratio in men [31, 35]. However, due to impossibility of the assessment of patient-individual risk factors in this study, it could not be conclusively determined how strong the influence on the study results was.

As previously mentioned, the NU/100-fractures ratio decreases from the age of 50–54 years onwards. A similar declining ratio pattern has also been described by Mills et al. [14]. This initially appears contradictory, as soft tissue quality and blood circulation tend to decline and risk factors like osteoporosis are more common in elderly [35]. However, higher percentage of low-energy fractures with increasing age results in less soft tissue damage and preservation of blood supply, which might contribute to lower NU/100-fractures ratios with increasing age. Another potential explanation for decreasing NU/fracture ratios could be the increase in overall and one-year mortality after fracture with increasing age [36]. According to Bergh et al., the one-year mortality rate after fracture increases to 1.4% (expected mortality: 0.4%) in individuals aged 50–64 years, while in those aged over 80 years, it reached 24.5% (expected mortality: 12.4%) [36]. In addition, perioperative mortality also increases with increasing age [37]. These high overall mortality and one-year post- and perioperative fracture-related mortality rates show that a remarkable percentage of elderly patients may die before NU can be diagnosed resulting in decreasing NU/100-fractures ratios. Moreover, NU occurrence was assessed using OPS-coded surgeries, which are performed only for symptomatic NUs eligible for surgical treatment. Factors like multimorbidity or immobility could result in asymptomatic NU or inability to surgery and therefore these undocumented NU could also contribute to lower NU numbers in this study. However, possible reasons for lower NU/100-fractures ratios with increasing age remain speculative as the analysed dataset does not provide information about individual mortality, morbidity and immobility.

This study based on routinely collected data demonstrates that the combination of ICD- and OPS-codes can be used to improve the resolution of anatomical localisation of NU. This methodological approach is easily transferable to other fields of research based on routine data of an entire health care system and can also be used in other countries relying on similar reimbursement coding systems. While using a population-level database and combining ICD- and OPS-codes provides an exceptionally high accuracy and eliminates sampling-related uncertainty, this methodological approach is also subject to important limitations, including the inability of individual case-tracking and assessment of effects of individual risk factors. Furthermore, potential coding inaccuracies should be considered.

Due to the clinically heterogeneous timing of NU diagnosis after fracture and the case-based structure of InEK data — organized by calendar year without longitudinal patient identifiers — fractures and surgically treated NU cases cannot be linked at the individual patient level. Consequently, the numerator (NU cases) and denominator (fracture cases) derive from separate cohorts recorded within the same calendar period. This methodological constraint precludes individual case-tracking and therefore evaluation of individual risk factors. Therefore, risk and incidence estimation was not possible, and analyses were limited to aggregated, calendar-year-based descriptive patterns at the population level. Although multi-year analysis reduces random variation, potential cohort mismatch bias cannot be fully eliminated. As a register-based study with limited available variables, several individual-level NU risk factors, which were reported in previous studies, could not be assessed in the present analysis. These include patient-related factors such as smoking status, diabetes mellitus, osteoporosis, and other comorbidities, as well as injury-related characteristics like trauma mechanism (high- vs. low-energy trauma), fracture severity, soft tissue damage, and fracture type (open vs. closed) [2, 3, 15, 31, 38]. Their potential contribution to observed variations in NU occurrence and NU/100-fractures ratios across anatomical regions, age groups, and sexes remains unevaluated, limiting result interpretability. Therefore, further studies are necessary to evaluate the impact of these risk factors on NU/fracture ratio and NU formation. The study period (2019–2023) spans the COVID-19 pandemic, during which fracture incidence changed due to altered population behaviour and healthcare utilisation. While the inclusion of multiple pre- and post-pandemic years mitigates abrupt temporal distortions, pandemic-related effects on fracture and NU patterns cannot be fully excluded [26, 39]. Finally, our methodology captured only surgically treated NU, as conservatively managed cases lack OPS-coding, which may lead to slight underestimation of overall NU occurrence. Conservatively managed NU of the femur and tibia are rare, as they must fulfil several fundamental prerequisites: sufficient mechanical stability, absence of malalignment or defects > 1 cm, biological activity, and infection exclusion [40]. As surgical treatment remains the therapeutic gold standard unsuccessfully treated conservative cases would eventually appear as surgical managed NU in our dataset. Despite these limitations, the study provides valuable insights into surgically treated NU of specific anatomical regions of the femur and the tibia.

## Conclusion

Using nationwide administrative data, this study provides an ICD- and OPS-based descriptive analysis of fracture and surgically treated NU incidences, as well as the surgically treated NU/100-fractures ratio of the tibia and femur in adults, with stratification by anatomical region, sex, and age. The observed NU/100-fractures ratios were consistently low relative to the overall fracture burden. However, these findings reflect surgically treated NU only and should not be interpreted as overall NU incidence. Marked variation in the NU/fracture ratio was observed between distinct anatomical regions within the same long bone. Proximal femur fractures, including femoral head and neck fractures, were associated with comparatively low NU/100-fractures ratios, whereas femoral shaft and distal femur fractures showed higher ratios. The highest NU/100-fractures ratios were observed for tibial shaft and distal tibia fractures.

The findings of this retrospective, population-based, cross-sectional register study highlight substantial anatomical heterogeneity in surgically treated NU compared to corresponding femur and tibia fractures and underline the impact of distinct anatomical localisations. We detected that independently of the anatomical localisation, men showed a higher NU/100-fractures ratio compared to women. With highest NU/fracture ratios in the economically active population, our study outlines the high socioeconomic burden and the need of further research about NU prevention and therapy.

## Data Availability

The data that support the findings of this study are available from the corresponding author upon reasonable request.

## Abbreviations

ICD: International Statistical Classification of Diseases and Related Health Problems
InEK: Institut für das Entgeltsystem im Krankenhaus (Institute for the Hospital Remuneration System)
DRG: Diagnosis-Related Groups
NU: Non-Union
OPS: Operation and Procedure Classification System
RR: Rate ratio
vs: versus
95 %-CI: 95 %-confidence interval
